# Long-term neurological health of the term offspring born via cesarean section for non-reassuring fetal monitoring

**DOI:** 10.1007/s00404-025-08258-2

**Published:** 2026-01-06

**Authors:** Gil Gutvirtz, Gali Pariente, Tamar Wainstock, Eyal Sheiner

**Affiliations:** 1 Department of Obstetrics and Gynecology, Soroka University Medical Center, Ben-Gurion University of the Negev, 151 Izak Rager Ave, 84101 Beer-Sheva, Israel; 2 Department of Public Health, Faculty of Health Sciences, Ben-Gurion University of the Negev, Beer-Sheva, Israel

**Keywords:** Cesarean delivery, Term delivery, Intrapartum, Fetal monitoring, Neurological morbidity, Perinatal outcomes, Long-term follow-up

## Abstract

**Purpose:**

While some evidence suggests that intrapartum fetal heart rate (FHR) monitoring is associated with a reduction in intrapartum death, a reduction in long-term neurological impairment has not been proven. In this study, we sought to evaluate the offspring long-term neurological morbidity of children born via cesarean delivery (CD) for non-reassuring FHR (NRFHR) indication.

**Methods:**

A population-based cohort analysis was performed comparing long-term neurological morbidity of term children born via CD for NRFHR as compared with children born via CD for non-progressive labor (NPL), at a single medical center. Neurological morbidity of the offspring was assessed using data from community-based clinics and/or hospitalizations up to 18 years involving neurological morbidity. A Kaplan–Meier survival curve was used to compare cumulative neurological morbidity incidence between the groups. A generalized estimating equations (GEE) model was used to control for possible confounders.

**Results:**

14,333 term singleton intrapartum CDs met the inclusion criteria. Of those, 59.0% were for NRFHR indication and 41.0% for NPL. Rate of total long-term neurological morbidity was comparable between the groups. The Kaplan–Meier survival curve also shows comparable cumulative incidence of neurological morbidity in both groups (Log-rank, *p* = 0.390). In the GEE model, controlling for repeated deliveries of the same mother (siblings), child birth year, follow-up time and multiple other confounders, NRFHR leading to CD was not found as a risk factor for offspring long-term neurological morbidity (aHR 1.87, 95%CI 0.74–4.72, *p* = 0.184).

**Conclusions:**

Intrapartum NRFHR leading to CD did not predict long-term neurological morbidity of the offspring, possibly due to prompt intervention.

**Supplementary Information:**

The online version contains supplementary material available at 10.1007/s00404-025-08258-2.

## Take-home message

**Table:** 

With timely intervention, non-reassuring fetal heart rate patterns that lead to emergent cesarean delivery do not appear to translate into adverse long-term neurological outcomes, including cerebral palsy. The findings are reassuring but should be interpreted within the limits of retrospective data and inherent subjectivity of FHR interpretation.	

## Introduction

Fetal heart rate (FHR) monitoring (cardiotocography-CTG) is the most common intrapartum surveillance method to trace fetal well-being during labor [1, 2]. It is an indirect marker of fetal cardiac and central nervous system responses to changes in blood pressure, blood gases, and acid–base status caused by uterine contractions. These changes are associated with inadequate fetal oxygenation that may predict a higher risk for fetal hypoxic injury or even death. The introduction of intrapartum FHR monitoring was meant to help obstetricians to identify fetuses at risk and enable a timely intervention to reduce the likelihood of perinatal adverse outcomes, as well as identify appropriately oxygenated fetuses and prevent unnecessary interventions. Unfortunately, the efficacy of intrapartum FHR monitoring for both purposes is still uncertain [3]. Although some evidence suggests that intrapartum fetal monitoring is associated with a reduction in intrapartum death [4], available data from trials comparing continuous electronic monitoring with intermittent auscultation failed to demonstrate that continuous monitoring was superior to intermittent auscultation with respect to preventing fetal death or poor long-term neurologic outcome. This was summarized in a meta-analysis [5] that reported no improvement in many fetal parameters including acidemia, Apgar scores, NICU admissions, hypoxic-ischemic encephalopathy and perinatal mortality. With regards to neurological outcomes, although fewer neonatal seizures were noted in the continuous monitoring group [6], the meta-analysis found no significant difference in rate of infant neurodevelopmental impairment at ≥ 12 months of age or cerebral palsy [5]. Importantly, in these trials, continuous monitoring was associated with more operative vaginal deliveries and cesarean sections for non-reassuring FHR patterns.

Newer methods of intrapartum surveillance that were introduced to better monitor the fetus, such as ST waveform analysis (STAN) [7], did not influence perinatal outcomes in several randomized controlled trials [8, 9], nor reduced the rate of emergency cesarean section [10]. A recent meta-analysis comparing multiple forms of intrapartum fetal monitoring found that no method of intrapartum fetal monitoring was associated with a reduced risk of neonatal acidemia, neonatal unit admissions, Apgar scores or perinatal death, questioning the effectiveness of intrapartum fetal monitoring in improving short-term neonatal outcomes [11].

There is an ongoing debate in the scientific literature regarding the long-term health implications for children born following abnormal fetal monitoring that led to CD [12, 13]. While some studies suggest a potential association between non-reassuring fetal heart rate patterns and future morbidity, others argue that these findings may be confounded by underlying conditions [12, 13].

This study is a large scale retrospective study, aimed to evaluate offspring long-term neurological morbidity following non-reassuring FHR (NRFHR) patterns during labor that necessitated an emergent (non-elective) cesarean delivery.

### Material and methods

This is a population-based retrospective cohort study, that included only term singleton cesarean deliveries (CD) of women in a single medical center, between 1991 and 2021. In our institution, we use continuous intrapartum FHR monitoring for virtually all women who enter active labor, whether their pregnancy is high or low risk.

We included only intrapartum (non-elective) cesarean deliveries. The exposure group was children born via CD due to NRFHR during the 1st and 2nd stage of labor, as compared with children born via CD due to labor dystocia (non-progressive labor (NPL) stage 1 or 2). All other indication for surgery such as non-vertex presentation, or placenta previa were excluded. This selection criteria enabled us to choose only cases of women in the active phase of labor that were assessed by a trained obstetrician who declared the indication for surgery. We excluded preterm deliveries as prematurity is strongly associated with adverse neurological outcomes. Also, we excluded all spontaneous and operative vaginal deliveries as these delivery modes constitute different exposure to the neonate. We also excluded multifetal pregnancies and fetuses with congenital malformations or chromosomal abnormalities as they are more prone and susceptible to neurological morbidity. Perinatal mortality cases were excluded from the long-term analysis.

The study was conducted at the Soroka University Medical Center (SUMC), the sole tertiary medical center in the Negev (southern Israel) and the largest birth center in the country. Thus, the study is based on non-selective population data. The institutional review board (SUMC IRB Committee) approved the study. Informed consent was not required as the data was anonymously coded.

The main outcome assed in this study was a diagnosis of a neurological morbidity of the offspring up to the age of 18 years, using ICD-9 diagnoses utilized in hospitalization records. A supplementary table containing the ICD-9 codes used (Table S1) further describes all diagnosis included under each neurological morbidity that was investigated in this study. Any hospitalization with a neurological morbidity was accounted for, meaning that even if the child was hospitalized for non-neurologic indication and had a background neurologic diagnosis that was previously coded in community-based clinics, it would have been counted as an event. Follow-up was terminated if any of the following occurred: first diagnosis of any of the neurological morbidities, death unrelated to neurological morbidity, end of the study period or when the child reached 18 years of age.

Secondary outcomes assessed were the perinatal outcomes of both study groups including gestational age at birth, birthweight, rates of low birthweight (LBW < 2500 g), low Apgar scores (< 7) and perinatal mortality.

Data were collected from 2 distinct computerized databases that were cross-linked based on the mother and offspring ID numbers: the perinatal database of the obstetrics and gynecology department which holds maternal demographic, obstetric and perinatal information and the pediatric hospitalization database of SUMC which encompass offspring demographic and hospitalization information for children hospitalized in SUMC pediatric departments. All diagnoses are based on ICD-9 codes for medical diagnoses used in the above mentioned databases.

### Statistical analysis

Statistical analysis was performed using the SPSS package 29th ed. (IBM/SPSS, Chicago, IL) and the STATA version 12.0. Categorical data are shown in counts and rates and the differences were assessed by chi-square for general associations. Student t-test was used for comparison of continuous variables with normal distribution. For the perinatal outcomes analysis, a generalized estimating equations (GEE) model was used to control for siblings (repeated deliveries of mothers) and potential confounders. Kaplan–Meier survival curves were used to compare cumulative hospitalization incidences over time among the study groups. Only the first diagnosis of a neurological condition for a given individual was included in the survival analysis. The differences between the curves were assessed using the log-rank test.

For the main outcome (long-term neurological morbidity), an additional GEE model was constructed to explore an association between NRFHR and future incidence of neurological morbidity of the offspring. The model was designed to account for siblings, follow up time and child birth year, while adjusting for possible confounding factors. These were chosen based on the univariate analysis as well as variables of clinical significance, and included: maternal age, gestational age, maternal hypertensive disorders of pregnancy (chronic hypertension, gestational or preeclampsia with or without severe features), maternal diabetes mellitus (either pre-gestational and gestational), induction of labor, oligohydramnios and low birthweight. All analyses were two-sided, and a *p*-value of ≤ 0.05 was considered statistically significant. ‬

## Results

During the study period, 14,333 term singleton cesarean deliveries met our inclusion criteria. 8455 (59.0%) were due to NRFHR and 5878 (41.0%) were due to labor dystocia (NPL). A participant flow diagram is available in the supplementary figure (Figure S1). Table 1 presents selected maternal characteristics. Women who had a CD due to NPL were older and more likely to be obese and diagnosed with diabetes (pre-gestational or gestational) and hypertensive disorders during pregnancy.

**Table 1:** Maternal characteristics of the study groups by the indication for CD

Maternal characteristic	CD d/t NRFHR N = 8455	CD d/t labor dystocia N = 5878	Odds ratio (OR)	95% Confidence interval	*p* value
Maternal age, years (mean ± SD)	28.4 ± 5.9	29.1 ± 5.9	–	–	< 0.001
Parity, n (%) 1 2–4 5 +	3874 (45.8%) 3031 (35.9%) 1549 (18.3%)	2730 (46.5%) 2206 (34.5%) 1121 (19.1%)			0.200
Obesity ^a^, n (%)	168 (2.0%)	194 (3.3%)			< 0.001
Fertility treatments, n (%)	405 (4.8%)	381 (6.5%)	0.72	0.62 – 0.83	< 0.001
Diabetes mellitus ^b^, n (%)	525 (6.2%)	626 (10.6%)	0.55	0.49 – 0.62	< 0.001
Hypertensive disorders ^c^, n (%)	571 (6.8%)	552 (9.4%)	0.69	0.61 – 0.79	< 0.001

^a^Defined as BMI > 30 kg/m^2^

^b^Including pre gestational and gestational diabetes

^c^Including pre gestational, gestational hypertension and pre-eclampsia

Table 2 summarizes pregnancy outcomes for both groups. Mean birth weight was significantly higher in the NPL group as well as mean gestational age. Rates of LBW and Low Apgar score at 5 min were higher in children in the NRFHR group. In the univariate analysis, perinatal mortality rates were significantly higher for children born via CD due to NRFHR. However, using a GEE model analyzing women as clusters to adjust for repeated deliveries of the same mother (siblings) and controlling for maternal age and gestational age, the association was no longer statistically significant (aOR = 12.6, 95% CI 0.09–1606.19, *p* = 0.30).

**Table 2:** Pregnancy outcomes of both study groups by the indication for CD

Pregnancy outcome	CD d/t NRFHR N = 8455	CD d/t labor dystocia N = 5878	Odds ratio (OR)	95% Confidence interval	*p* value
Oligohydramnios, n (%)	585 (6.9%)	210 (3.6%)	2.01	1.70 – 2.35	< 0.001
Induction of labor, n (%)	3,045 (36.0%)	3,054 (52.0%)	0.52	0.48 – 0.55	< 0.001
Gestational age at birth, weeks (mean ± SD)	39.6 ± 1.3	39.7 ± 1.3	–	–	< 0.001
Birthweight, grams (mean ± SD)	3124 ± 507	3434 ± 471	–	–	< 0.001
Low birthweight (< 2500 g), n (%)	882 (10.4%)	161 (2.7%)	4.13	3.48 – 4.90	< 0.001
Low Apgar (< 7) at 5 min, n (%)	284 (3.4%)	82 (1.4%)	2.46	1.92 – 3.15	< 0.001
Perinatal mortality, n (%)	91 (1.1%)	21 (0.4%)	3.03	1.88 – 4.88	< 0.001

Excluding eating disorders, the rates of long-term neurological morbidities were all comparable between groups (presented in Table 3), as well as the total neurological morbidity rate. Eating disorders were more prevalent in the NPL group as compared to the NRFHR group.

**Table 3:** Selected long-term neurological morbidities in children (up to the age of 18 years) born via CD due to NRFHR as compared to children born via CD due to labor dystocia (non-progressive labor)

Neurological morbidity	CD d/t NRFHR N = 8455	CD d/t labor dystocia N = 5878	Odds ratio (OR)	95% Confidence interval	*p* value
Autism, n (%)	11 (0.1%)	8 (0.1%)	0.95	0.38 – 2.37	0.923
Eating disorders, n (%)	32 (0.4%)	39 (0.7%)	0.57	0.35 – 0.90	0.017
Movement disorders, n (%)	290 (3.4%)	207 (3.5%)	0.97	0.81 – 1.16	0.768
Cerebral palsy, n (%)	39 (0.5%)	15 (0.3%)	1.81	0.99 – 3.29	0.052
Developmental disorders, n (%)	101 (1.2%)	58 (1.0%)	1.21	0.87 – 1.67	0.243
Degenerative disorders, n (%)	23 (0.3%)	23 (0.4%)	0.69	0.38 – 1.23	0.214
Myopathy, n (%)	18 (0.2%)	15 (0.3%)	0.83	0.42 – 1.65	0.603
**Total neurological morbidity, n (%)**	**702 (8.3%)**	**516 (8.8%)**	**0.94**	**0.83 – 1.06**	**0.315**

Bold values indicate the main outcomes

**Fig. 1:**
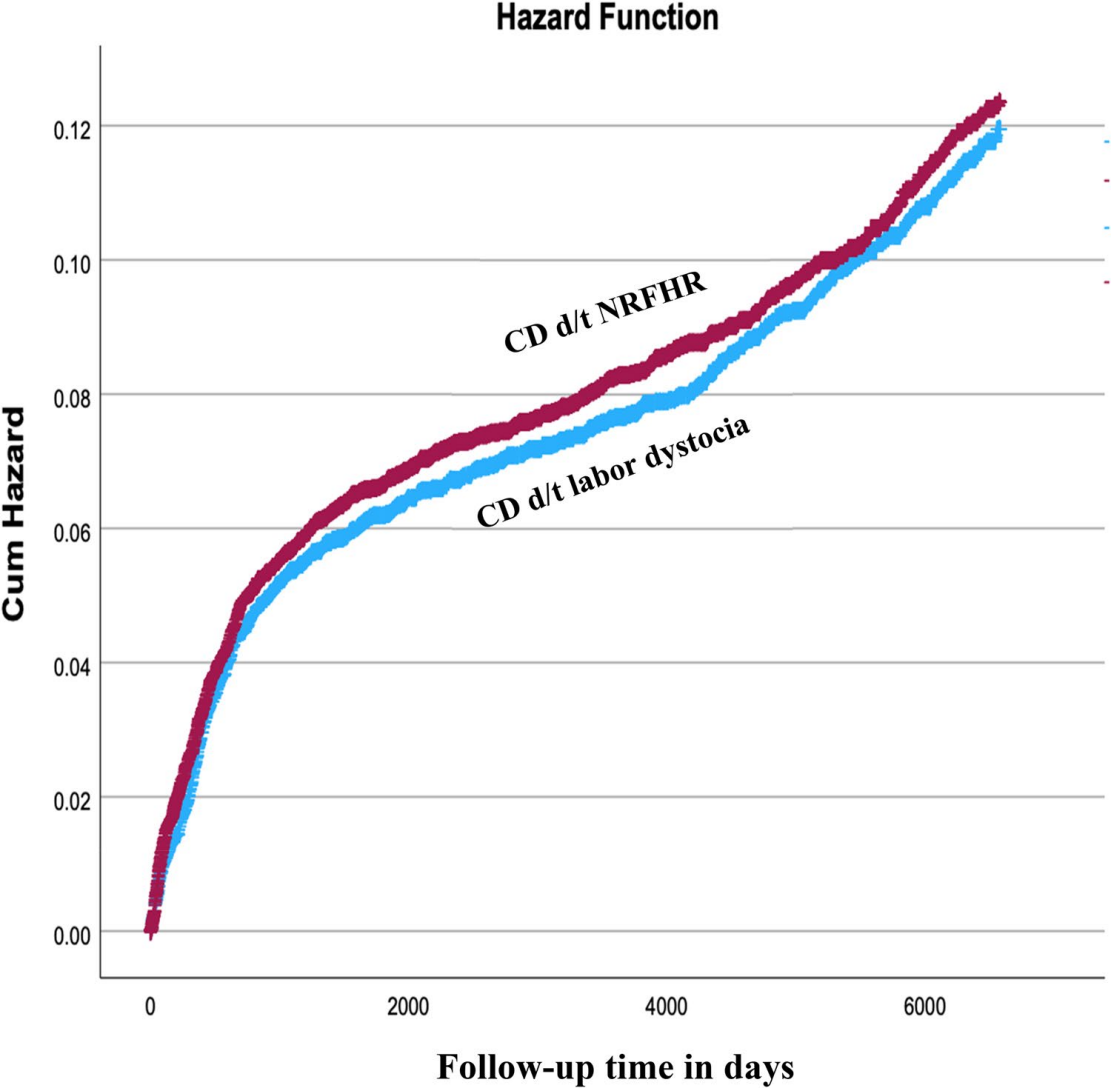
Kaplan–Meier survival curve demonstrating the cumulative incidence of neurological morbidity in children born via CD due to NRFHR and children born via CD due to labor dystocia (Log-rank, *p* = 0.390)

The Kaplan–Meier survival curve (shown in Fig. 1), demonstrated comparable cumulative incidence of neurological morbidity between the NRFHR and NPL groups (Log-rank, *p* = 0.390). Table 4 presents the GEE model used for the association between long-term risk for neurological morbidity in children (up to the age of 18 years) and the NRFHR indication for CD. The model adjusts for maternal age, gestational age, maternal diabetes and hypertensive disorders, induction of labor, oligohydramnios and LBW neonates. It also takes into account repeated deliveries of the same mother (siblings), the length of follow up for the offspring and the child’s birth year. In this multivariable model, NRFHR pattern leading to intrapartum CD was not associated with adverse long-term neurological outcomes (aHR 1.87, 95% CI 0.74–4.72, *p* = 0.184).

**Table 4:** GEE model* for the association between NRFHR monitoring and neurological morbidity of the offspring

	Hazard ratio	95% CI	*p* value
**Non-reassuring FHR**	**1.87**	**0.74 – 4.72**	**0.184**
Maternal age	1.05	1.00 – 1.10	0.047
Gestational age	0.92	0.74 – 1.15	0.488
Hypertensive disorders	1.19	0.89 – 1.59	0.227
Diabetes mellitus	0.91	0.61 – 1.36	0.668
Child birth year	0.58	0.48 – 0.71	< 0.001
Low birth weight (< 2500 g.)	0.82	0.20 – 3.27	0.785
Induction of labor	1.06	0.56 – 1.99	0.848
Oligohydramnios	1.03	0.74 – 1.44	0.827

Bold values indicate the main outcomes

The model also accounts for repeated deliveries of the same mother and offspring follow-up time

## Discussion

The main finding of our study is that NRFHR patterns leading to emergent cesarean delivery during labor, were not associated with significant adverse perinatal outcomes nor long-term neurological morbidity, including cerebral palsy (CP).

While this lack of association is somewhat reassuring, it does not imply an evidence for no effect and the results should be interpreted in the frame of a retrospective study with its inherent flaws that will be discussed here.

Since the introduction of intrapartum fetal monitoring, numerous trials aimed to provide evidence of its efficacy to improve perinatal outcomes. A 1995 meta-analysis by Vintzileos et al. [4] found that continuous monitoring decreased perinatal mortality on the expanse of higher rates of operative and cesarean deliveries. However, later Cochrane reviews and meta-analysis of more recent trials found that continuous monitoring showed no significant improvement in overall perinatal death rate [5, 14–16], still inflicting higher risk for surgical interventions.

Importantly, the Cochrane review also acknowledges that most outcomes assessed in the included trials were graded as low quality evidence with a high risk for biases.

For obvious reasons, no randomized trials have compared intrapartum fetal monitoring with no intrapartum fetal monitoring as such a trial would be unethical.

While the positive predictive value of NRFHR is considered low, it still proves high specificity and negative predictive value [17, 18] in both low and high-risk pregnancies. When Tarvonen et al. investigated 314 cases of hypoxic-ischemic encephalopathy (HIE) in term neonates, they found that nearly half (44.9%) presented a normal admission CTG, indicating that this outcome probably had intrapartum onset. After excluding sentinel events and timely deliveries, about half of intrapartum HIE cases were deemed potentially avoidable with better intrapartum care [19]. The potential of fetal monitoring to improve perinatal outcomes has led national organizations to advise continuous CTG for high-risk pregnancies (e.g., preeclampsia or suspected growth restriction etc.) and complicated births (suspected chorioamnionitis, meconium etc.) [20, 21].

Our study revealed higher perinatal death rates in the univariate analysis for the NRFHR group, which is in accordance with earlier reports, but we were careful to conduct a GEE model to control for possible confounders and did found that when controlling for repeated deliveries of the same mother and other crucial clinical variables such as maternal and gestational age, this association was no longer statistically significant.

Regarding neurological morbidity and CP, the latest Cochrane [5] included 9 trials that together found continuous fetal monitoring halved neonatal seizure rates (RR 0.50, 95% CI 0.31 to 0.80), but no difference was found in CP rates (2 trials).

One interesting trial from 1996 published in NEJM did found a correlation between specific abnormal findings on FHR monitoring and an increased risk of cerebral palsy, but the false positive rate (abnormal patterns in FHR monitoring and no CP) was extremely high (99.8%), leading the authors to admit that if CD is indicated for these abnormal patterns many cesarean sections would be performed without neonatal benefit and with the potential for harm to the mother [22].

Whether the reduction seen in neonatal seizures is something inconsequential that should not greatly influence women's and clinicians' choices, or if seizure reduction leads to long‐term benefits for babies is still unclear. Studies have shown that non-reassuring patterns of FHR during labor correlate with fetal acidosis at birth [23–27], but even if NRFHR monitor could predict perinatal hypoxia/ischemia it is postulated that acute intrapartum hypoxic event accounts for only a small minority of cases of CP [28–32], even as low as 1% [33, 34]. Furthermore, the degree of fetal oxygen deprivation that leads to long-term neurologic injury is close to that causing fetal death; thus, many severely depressed fetuses may either survive intact or die, rather than survive with disability [24].

Our results show that NRFHR patterns did not predict long-term neurological damage for the offspring, which is reassuring. However, even when no association is found, it does not imply equivalence of the groups and NRFHR is not a benign scenario. The complexity of predicting long-term outcomes based solely on intrapartum findings, underscores the need for cautious interpretation of FHR monitoring data.

One plausible explanation is that the decision to expedite delivery by CD may have discontinued fetal oxygen deprivation during labor and may have contributed to prevent consequent neurological damage. Although it is practically impossible to prove in a randomized clinical trial, it is plausible to assume that good recognition of abnormal FHR patterns and promptly applying appropriate therapeutic interventions may lead to better neonatal outcomes [35, 36], and possibly result in comparable neurological outcomes with children born due to labor dystocia.

In this study, we excluded elective CDs which, by definition, are those who are not suitable for a trial of vaginal delivery and as such are delivered prior to labor initiation. Previous trials have shown that elective cesarean delivery is associated with offspring long-term adverse consequences as compared with vaginal deliveries, including increased risk for cardiovascular [37], respiratory [38], infectious [39], endo-metabolic [40] and specifically neurologic [41] morbidity.

We also decided to exclude vaginal operative deliveries as this mode of delivery differs significantly from CD and introduces distinct exposure to the offspring. However, we acknowledge that this exclusion may omit the most time-critical NRFHR cases where operative vaginal delivery was the most expedient intervention, so we conducted a secondary analysis of operative vaginal deliveries comparing those to CD for NRFHR and found no statistical significant difference in the long-term incidence of neurological morbidity (Kaplan–Meier, Log rank *p* = 0.056, Supplementary Figure S1). This is further supported by a study of Ulfsdottir et al. that compared long-term neurological morbidity of offspring delivered by vaccum extraction as compared with emergency cesarean deliveries [42].

Intrapartum FHR monitoring was meant to identify fetal behavior and fetal responses to the mechanical and hypoxemic stresses of contractions, so the comparison of CDs due to NRFHR with CDs for labor dystocia was meant to differentiate between intrapartum scenarios where the fetal monitor may indicate fetal distress in labor to scenarios where the CD indication is for non-progressive labor, presumably, without the suspicion of fetal distress in labor. By choosing these groups we were investigating whether possible intrapartum insults represented by fetal monitoring would result in long-term neurological morbidity, and no such association was found.

The higher rate of eating disorders observed in the dystocia group is an intriguing finding, though its interpretation is limited. Extensive research of the literature found no established relationship between labor dystocia and eating disorders in offspring. Also, eating disorders were the only neurological outcome showing a statistically significant difference, and the base rate was very low (0.4 vs. 0.7%), so this finding possibly reflects a chance finding. Given the multifactorial etiology of eating disorders and the retrospective nature of our study, this association should be interpreted cautiously and may warrant further investigation in dedicated prospective cohorts.

The study's main strength lies in its non-selective population based relatively large cohort that enabled reaching statistical results for even rare events, such as pediatric neurological morbidities. Also, we used only intrapartum CDs that enabled us to define an appropriate control group and exclude other delivery modes and complications. Furthermore, SUMC serves as the largest birth center in Israel and is the sole tertiary hospital in the region. This allows for a unique opportunity to combine maternal and offspring data using combined perinatal and pediatric hospitalization records, enabling an accurate follow-up of children born in our institution. Since SUMC is the only tertiary center, most offspring that are born in SUMC are expected also to be hospitalized in the same institution if needed. It is possible that some women sought medical care for their child outside the region, introducing a potential ascertainment bias. However, there is no apparent reason to believe that this behavior differed between the comparison groups.

While the long follow-up period of the study is one of its major strengths, evolution in obstetric management, neonatal care, and diagnostic definitions of neurodevelopmental disorders may have affected both exposure classification and outcome ascertainment during this period. In order to overcome part of this limitation, we applied a multivariable model that incorporates offspring year of birth and follow-up time to account for possible temporal changes in practice that might have evolved during the study period.

Also, our study has other limitations that should be noted and discussed. The first being the retrospective design of the study, as data was collected through computerized databases, where the indications for cesarean delivery were recorded.

As such, original FHR tracings and partograms for the definition of normal or abnormal labor progress were not consistently archived and thus not available for review. We acknowledge that these clinical scenarios are not mutually exclusive and often involve overlapping indications. Importantly, fetal monitoring interpretation is observer dependent [43, 44], and to date, there are no recommended adjunct modalities for the interpretation of FHR monitoring [45] to assist physician in clinical decision making. Although the new classification of intrapartum monitoring to 3 distinct categories (the three-tier FHR classification system) [21] allows better definition of reassuring (category 1) and non-reassuring (category 3) fetal monitor, most intrapartum monitors are classified as category 2 which leaves wide margins for personal interpretation of the fetal heart rate tracing. While some will define a category 2 fetal monitoring as approving continuous vaginal delivery, others might consider the same FHR tracing as non-reassuring which calls for an emergent CD [46]. However, the same may be argued for the definitions of non-progressive labor. As such, both groups might have suffered “observer” dependency which also limits comparability across decades.

Also, a healthy neurological development of children is closely associated with many genetic, socioeconomic and environmental factors, many of them are virtually impossible to capture in a retrospective study. These factors may independently or interactively modify fetal vulnerability to labor-related insults, and their unmeasured presence may confound associations between intrapartum factors and long-term outcomes. Nevertheless, as far as genetics are involved, we carefully adjusted for recurrent deliveries of the same mother, in attempt to counter any genetic differences among siblings.

In conclusion, although the efficacy of intrapartum FHR monitoring to improve perinatal or long-term offspring outcomes has not been proven, it continues to be the most common method for fetal surveillance during labor. While some will argue that its continuous use might actually lead to unnecessary interventions to expedite delivery, it is unlikely that intrapartum FHR monitoring will be abandoned since normal FHR tracing gives reassurance for fetal well-being and virtually excludes primary intrapartum hypoxia.

Our study found that NRFHR indication for CD did not affect the offspring neurological health, and was not related to higher rates of CP in childhood or adolescence, most probably due to prompt intervention by the obstetric teams. These results underscore the fact that survival and neonatal outcome is highly dependent on timing and mode of intervention following a prenatal diagnosis of NRFHR. As we used only CDs in our study, we cannot draw any conclusions regarding the use of intrapartum FHR monitoring and its influence on the obstetrician considering the appropriate delivery mode. Better techniques for intrapartum fetal assessment are still required that may eventually enable a better and safer management of deliveries.

## Supplementary Information

Below is the link to the electronic supplementary material.

Supplementary file1 (PDF 50 KB)

Supplementary file2 (DOCX 27 KB)

## Data Availability

No datasets were generated or analysed during the current study.
