# Supplementary figures and images for: Long-term neurological health of the term offspring born via cesarean section for non-reassuring fetal monitoring
Source: Arch Gynecol Obstet. 2026 Jan 6;313(1):13. doi: 10.1007/s00404-025-08258-2 (PMC12774927; doi:10.1007/s00404-025-08258-2)

## Participant Flow Diagram

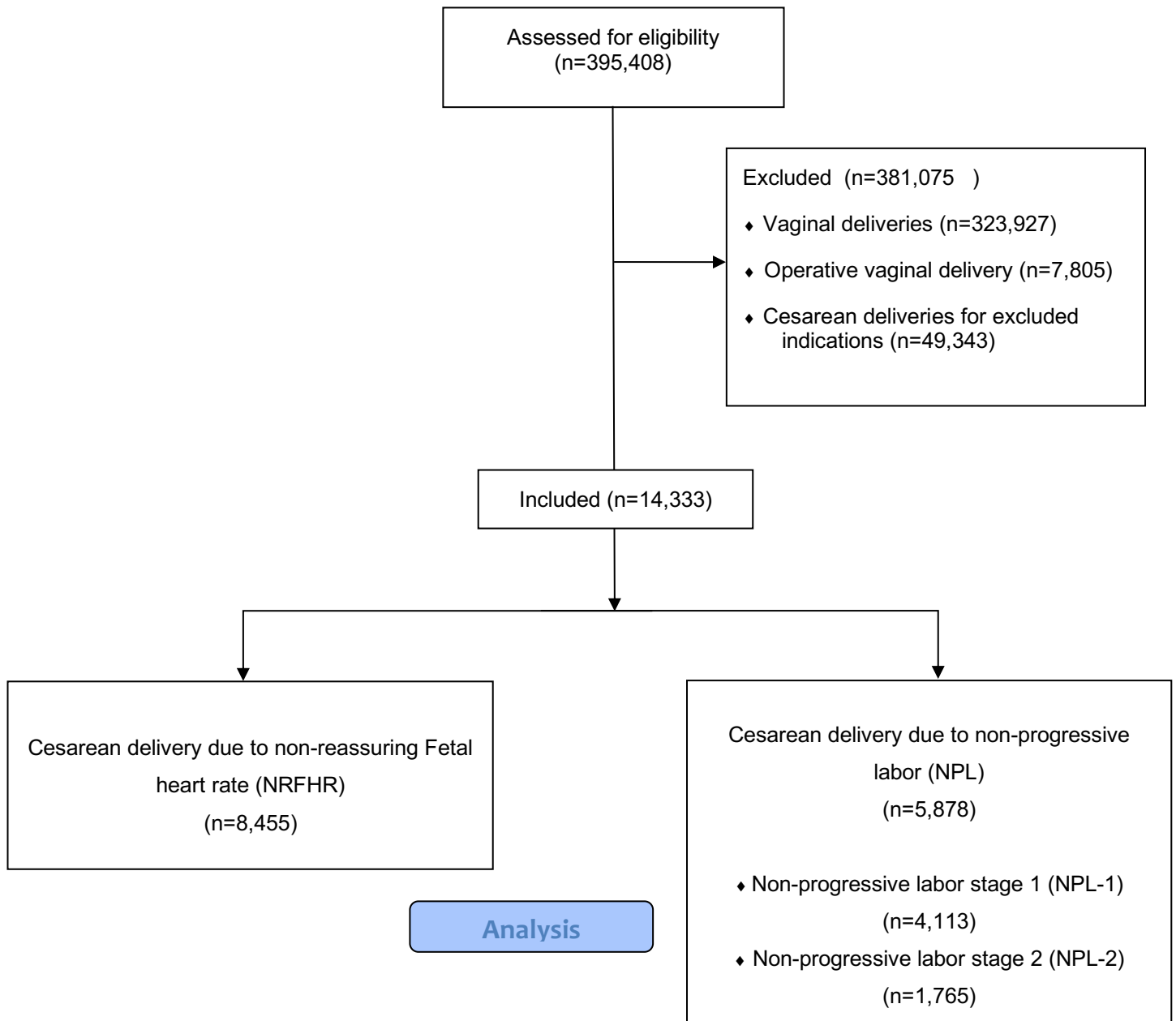

Supplement: Supplementary file 1 — Supplementary file1 (PDF 50 KB) [file 404_2025_8258_MOESM1_ESM.pdf]
