# Supplementary material for: Long-term neurological health of the term offspring born via cesarean section for non-reassuring fetal monitoring
Source: Arch Gynecol Obstet. 2026 Jan 6;313(1):13. doi: 10.1007/s00404-025-08258-2 (PMC12774927; doi:10.1007/s00404-025-08258-2)
Supplement: Supplementary file 2 — Supplementary file2 (DOCX 27 KB) [file 404_2025_8258_MOESM2_ESM.docx]

**Supplementary Table – ICD-9 Codes of neurological morbidity**

| **Group** | **Diag. Code** | **Diagnosis Description** |
| --- | --- | --- |
| **AUTISM** | 2990 | AUTISTIC DISORDER |
|  | 2990 | INFANTILE AUTISM |
|  | 2998 | OTHER SPECIFIED PERVASIVE DEVELOPMENTAL DISORDERS |
|  | 29900 | AUTISTIC DISORDER, CURRENT OR ACTIVE STATE |
|  | 29901 | AUTISTIC DISORDER, RESIDUAL STATE |
|  | 29910 | CHILDHOOD DISINTEGRATIVE DISORDER, CURRENT OR ACTIVE STATE |
|  | 29981 | OTHER SPECIFIED PERVASIVE DEVELOPMENTAL DISORDERS, RESIDULA STATE |
|  | 29990 | UNSPECIF.PERVASIVE DEVELOPMENTAL DISORDER, CURRENT OR ACTIVE STATE |
| **EATING DISORDERS** | 3071 | ANOREXIA NERVOSA |
|  | 3075 | OTHER AND UNSPECIFIED DISORDERS OF EATING |
|  | 30750 | EATING DISORDER, UNSPECIFIED |
|  | 30751 | BULIMIA NERVOSA |
|  | 30753 | RUMINATION DISORDER |
|  | 30759 | OTHER DISORDERS OF EATING |
|  | V691 | INAPPROPRIATE DIET & EATING HABITS |
| **MOVEMENT DISORDERS** | 3073 | STEREOTYPIC MOVEMENT DISORDER |
|  | 3331 | ESSENTIAL AND OTHER SPECIFIED FORMS OF TREMOR |
|  | 3332 | MYOCLONUS |
|  | 3335 | OTHER CHOREAS |
|  | 3336 | GENETIC TORSION DYSTONIA |
|  | 3336 | IDIOPATHIC TORSION DYSTONIA |
|  | 3343 | OTHER CEREBELLAR ATAXIA |
|  | 3450 | GENERALIZED NONCONVULSIVE EPILEPSY |
|  | 3452 | PETIT MAL STATUS, EPILEPTIC |
|  | 3453 | GRAND MAL STATUS, EPILEPTIC |
|  | 3455 | PARTIAL EPILEPSY, WITHOUT IMPAIRMENT OF CONSCIOUSNESS |
|  | 3456 | INFANTILE SPASMS |
|  | 3459 | EPILEPSY, UNSPECIFIED |
|  | 7810 | ABNORMAL INVOLUNTARY MOVEMENTS |
|  | 7812 | ABNORMALITY OF GAIT |
|  | 7813 | LACK OF COORDINATION |
|  | 33390 | UNSP.EXTRAPYRAMIDAL DISEASE + ABNORMAL MOVEMENT DISORDER |
|  | 33399 | OTHER EXTRAPYRAMIDAL DISEASES AND ABNORMAL MOVEMENT DISORDERS |
|  | 34500 | GENERALIZED NONCONVULSIVE EPILEPSY WITHOUT INTRACTABLE EPILEPSY |
|  | 34501 | GENERALIZED NONCONVULSIVE EPILEPSY WITH INTRACTABLE EPILEPSY |
|  | 34510 | GENERALIZED CONVULSIVE EPILEPSY WITHOUT INTRACTABLE EPILEPSY |
|  | 34511 | GENERALIZED CONVULSIVE EPILEPSY WITH INTRACTABLE EPILEPSY |
|  | 34540 | PARTIAL EPILEPSY+IMPAIRMENT OF CONSCIOUSNESS WITHOUT INTRACTABLE EPILEPSY |
|  | 34550 | PARTIAL EPILEPSY WITHOUT IMPAIRMENT OF CONSCIOUSNESS WITHOUT INTR ACTABEL EPILEPSY |
|  | 34560 | INFANTILE SPASMS WITHOUT INTRACTABLE EPILEPSY |
|  | 34590 | EPILEPSY, NUSP. WITHOUT INTRACTABEL EPILEPSY |
|  | 34590 | EPILEPSY, UNSP. WITHOUT INTRACTABLE EPILEPSY |
|  | 34591 | EPILEPSY UNSP. WITH INTRACTABLE EPILEPSY |
|  | 78031 | FEBRILE CONVULSIONS |
|  | 78031 | FEBRILE CONVULSIONS (SIMPLE), UNSPECIFIED |
|  | 78032 | COMPLEX FEBRILE CONVULSIONS |
|  | 78039 | OTHER CONVULSIONS |
|  | 78099 | OTHER GENERAL SYMPTOMS |
| **CEREBRAL PALSY** | 3341 | HEREDITARY SPASTIC PARAPLEGIA |
|  | 3421 | SPASTIC HEMIPLEGIA |
|  | 3429 | HEMIPLEGIA, UNSPECIFIED |
|  | 3430 | CONGENITAL DIPLEGIA |
|  | 3431 | CONGENITAL HEMIPLEGIA |
|  | 3432 | CONGENITAL QUADRIPLEGIA |
|  | 3439 | INFANTILE CEREBRAL PALSY, UNSPECIFIED |
|  | 3441 | PARAPLEGIA |
|  | 3442 | DIPLEGIA OF UPPER LIMBS |
|  | 3449 | PARALYSIS, UNSPECIFIED |
|  | 3481 | ANOXIC BRAIN DAMAGE |
|  | 3526 | MULTIPLE CRANIAL NERVE PALSIES |
|  | 7814 | TRANSIENT PARALYSIS OF LIMB |
|  | 34210 | SPASTIC HEMIPLEGIA AFFECTING UNSP. SIDE |
|  | 34290 | HEMIPLEGIA, UNSP., AFFECTING UNSP. SIDE |
|  | 34291 | HEMIPLEGIA, UNSP., AFFECTING DOMINANT SIDE |
|  | 34292 | HEMIPLEGIA, UNSP., AFFECTING NONDOMINANT SIDE |
|  | 34400 | QUADRIPLEGIA, UNSPECIFIED |
|  | 34430 | MONOPLEGIA OF LOWER LIMB, AFFECTING UNSP. SIDE |
|  | 34440 | MONOPLEGIA OF UPPER LIMB, AFFECTING UNSP. SIDE |
|  | 34489 | OTHER SPECIFIED PARALYTIC SYNDROME |
|  | 43811 | APHASIA |
|  | 43820 | HEMIPLEGIA AFFECTING UNSP. SIDE |
| **DEVELOPMENTAL DISORDERS** | 317 | MILD INTELLECUTAL DISABILITIES |
|  | 317 | MILD MENTAL RETARDATION |
|  | 319 | UNSPECIFIED INTELLECTUAL DISABILITIES |
|  | 319 | UNSPECIFIED MENTAL RETARDATION |
|  | 3152 | OTHER SPECIFIC DEVELOPMENTAL LEARNING DIFFICULTIES |
|  | 3154 | DEVELOPMENTAL COORDINATION DISORDER |
|  | 3158 | OTHER SPECIFIED DELAYS IN DEVELOPMENT |
|  | 3159 | UNSPECIFIED DELAY IN DEVELOPMENT |
|  | 7834 | LACK OF EXPECTED NORMAL PHYSIOLOGICAL DEVELOPMENT |
|  | 7834 | LACK OF EXPECTED NORMAL PHYSIOLOGICAL DEVELOPMENT IN CHILDHOOD |
|  | 31531 | EXPRESSIVE LANGUAGE DISORDER |
|  | 31534 | SPEECH AND LANGUAGE DEVELOPMENTAL DELAY DUE TO HEARING LOSS |
|  | 31539 | OTHER DEVELOPMENTAL SPEECH DISORDER |
|  | 33183 | MILD COGNITIVE IMPAIRMENT, SO STATED |
|  | 78340 | LACK OF NORMAL PHYSIOLOGICAL DEVELOPMENT, UNSPECIFIED |
| **DEGENERATIVE DISORDERS** | 330 | CEREBRAL DEGENERATIONS USUALLY MANIFEST IN CHILDHOOD |
|  | 335 | ANTERIOR HORN CELL DISEASE |
|  | 340 | MULTIPLE SCLEROSIS |
|  | 3300 | LEUKODYSTROPHY |
|  | 3308 | OTHER SPECIFIED CEREBRAL DEGENERATIONS IN CHILDHOOD |
|  | 3313 | COMMUNICATING HYDROCEPHALUS |
|  | 3314 | OBSTRUCTIVE HYDROCEPHALUS |
|  | 3319 | CEREBRAL DEGENERATION, UNSPECIFIED |
|  | 3348 | OTHER SPINOCEREBELLAR DISEASES |
|  | 3350 | WERDNIG-HOFFMANN DISEASE |
|  | 3360 | SYRINGOMYELIA AND SYRINGOBULBIA |
|  | 3410 | NEUROMYELITIS OPTICA |
|  | 3411 | SCHILDER'S DISEASE |
|  | 3419 | DEMYELINATING DISEASE OF CENTRAL NERVOUS SYSTEM, UNSPECIFIED |
|  | 3480 | CEREBRAL CYSTS |
|  | 3590 | CONGENITAL HEREDITARY MUSCULAR DYSTROPHY |
|  | 3591 | HEREDITARY PROGRESSIVE MUSCULAR DYSTROPHY |
|  | 33189 | OTHER CEREBRAL DEGENERATION |
|  | 33510 | SPINAL MUSCULAR ATROPHY, UNSPECIFIED |
|  | 33522 | PROGRESSIVE BULBAR PALSY |
|  | 33523 | PSEUDOBULBAR PALSY |
|  | 34120 | ACUTE (TRANSVERSE) MYELITIS NOS |
|  | 348891 | CEREBRAL CALCIFICATION |
|  | 3313 2 | POST HEMORRHAGIC HYDROCEPHALUS |
| **MYOPATHIES** | 352 | DISORDERS OF OTHER CRANIAL NERVES |
|  | 3379 | UNSPECIFIED DISORDER OF AUTONOMIC NERVOUS SYSTEM |
|  | 3510 | BELL'S PALSY |
|  | 3518 | OTHER FACIAL NERVE DISORDERS |
|  | 3519 | FACIAL NERVE DISORDER, UNSPECIFIED |
|  | 3539 | UNSPECIFIED NERVE ROOT AND PLEXUS DISORDER |
|  | 3542 | LESION OF ULNAR NERVE |
|  | 3548 | OTHER MONONEURITIS OF UPPER LIMB |
|  | 3549 | MONONEURITIS OF UPPER LIMB, UNSPECIFIED |
|  | 3553 | LESION OF LATERAL POPLITEAL NERVE |
|  | 3556 | LESION OF PLANTAR NERVE |
|  | 3558 | MONONEURITIS OF LOWER LIMB, UNSPECIFIED |
|  | 3559 | MONONEURITIS OF UNSPECIFIED SITE |
|  | 3562 | HEREDITARY SENSORY NEUROPATHY |
|  | 3564 | IDIOPATHIC PROGRESSIVE POLYNEUROPATHY |
|  | 3568 | OTHER SPECIFIED IDIOPATHIC PERIPHERAL NEUROPATHY |
|  | 3569 | UNSPECIFIED IDIOPATHIC PERIPHERAL NEUROPATHY |
|  | 3570 | ACUTE INFECTIVE POLYNEURITIS |
|  | 3571 | POLYNEUROPATHY IN COLLAGEN VASCULAR DISEASE |
|  | 3572 | POLYNEUROPATHY IN DIABETES |
|  | 3577 | POLYNEUROPATHY DUE TO OTHER TOXIC AGENTS |
|  | 3588 | OTHER SPECIFIED MYONEURAL DISORDERS |
|  | 3589 | MYONEURAL DISORDERS, UNSPECIFIED |
|  | 3592 | MYOTONIC DISORDERS |
|  | 3599 | MYOPATHY, UNSPECIFIED |
|  | 33709 | OTHER IDIOPATHIC PERIPHERAL AUTONOMIC NEUROPATHY |
|  | 33720 | REFLEX SYMPATHETIC DYSTROPHY, UNSPECIFIED |
|  | 33721 | REFLEX SYMPATHETIC DYSTROPHY OF UPPER LIMB |
|  | 33722 | REFLEX SYMPATHETIC DYSTROPHY OF LOWER LIMB |
|  | 35781 | CHRONIC INFLAMMATORY DEMYELINATING POLYNEURITIS |
|  | 35800 | MYASTHENIA GRAVIS WITHOUT (ACUTE) EXACERBATION |
